# Modus Operandi of Poisons

**Published:** 1829

**Authors:** 


34. *Modus operandi of Poisons.* Believing, as we do, that the *modus operandi* of medicines and poisons is the same; and that from the greater energy and more distinct impression of the latter, the study of their effects may throw light upon the action of medicinal agents, we perceive in well conducted toxicological experiments, many important advantages to therapeutic medicine. The last number of the Medico-Chirurgical Review, (July, 1829,) contains an interesting review of an experimental and theoretical essay on this subject by Dr. Addison and Mr. Morgan, two practitioners of the British metropolis. As the essay has not reached us, we shall extract such parts as will give our readers an idea of their experiments, and the conclusions at which they have arrived.

We have, say the reviewers, already given the theory of Dr. Addison and Mr. Morgan, namely, that, "all poisons act on the system through the medium of nerves only." They are surprised that former physiologists should not have admitted this theory. They had seen it exemplified in the effect of opium on a pained part, of belladonna on the iris, and in certain phenomena which take place in disease. Thus—

"A person receives a slight lacerated wound, a burn, a puncture from a spicula of wood, or a rusty nail; the irritating cause being removed from the part, all shall appear to be going on well, when suddenly symptoms of tetanus supervene, and proceed to the destruction of life: here we have, then, the mere irritation of the nerves of a small portion of the body, so deranging and involving the entire nervous system, as to give rise to one of the most formidable of all diseases, and this, so far as we know, without the slightest evidence of any thing noxious being directly applied to the brain. If, then, mere irritation, mechanical or otherwise, prove sufficient to derange the whole nervous system in this instance, where, we would ask, is the difficulty of conceiving that morbid irritation, and consequent general derangement, in the system, should result from the application of a poison, altogether independently of any absorption whatever?"—p. 65.

These are strong facts in support of the proposed theory. Again we are reminded how many poisons act too suddenly for the medium of absorption. It is said, that the exclusion of the sciatic nerve in a limb included in a tight ligature as already described, rendered that nerve incapable of performing its functions in the absence of all circulation. This is a strong objection, but one that equally applies to the experiments of our authors. Of this hereafter.

In confirmation of our authors' deductions the following experiments were instituted.

"To prove the extreme susceptibility of the inner coat of a vein, when exposed to action of a poison, the following experiments were made:—

"The jugular vein of a full grown dog (in size about that of a common harrier) was laid bare to the extent of about two inches; the circulation through the denuded vessel was then completely stopped by the application of two temporary ligatures, one of which was tied round the upper, and the other round the lower, part of the exposed vein; the vessel was then divided between the two ligatures, and the truncated extremities reconnected by means of a short brass cylinder or tube, within which was placed a portion of woorara, of the size of a grain of canary seed. In this way the continuity of the canal between the temporary ligature was preserved entire, the brass tube being inserted and tied within the mouths of the cut extremities, and consequently, allowing, on the previous impediment to circulation being removed, a free passage of blood from the upper to the lower part of the vein; with which blood, of course, the poison in the interior of the tube would instantly be mixed, and carried through the circulation.

"Both the temporary ligatures being then removed, the accustomed circulation through the vessel was re-established; and in forty-five seconds the animal dropped on the ground, completely deprived of all power over the muscles of voluntary motion; in two minutes convulsions and respiration had entirely ceased.

"We were perfectly aware, in making this experiment, that the result might be adduced in proof of the truth of the cerebral contact, as well as of nervous communication of a poison; it was therefore, merely made for the purpose of affording a contrast to the following:

"The jugular vein of a dog, of the same size and age of the preceding, (both being from the same litter,) was exposed, and separated from its surrounding connexion, to the extent of three inches; temporary ligatures were then applied, as in the former case. A small opening was then made in the vein, immediately above the lower ligature, through which a cylinder of quill was pushed into the interior of the vessel; within this quill a piece of woorara, of the same size as used before, had been previously inserted. The vein was now again tied by a permanent ligature above the opening, through which the quill had been thrust, leaving a space of two inches and three quarters between the first upper temporary ligature and the permanent one last mentioned; the interior of the vessel between the two containing the poison which was thus prevented from coming in contact with the sides of the vessel, until washed by blood out of the quill in which it was contained. The upper temporary ligature was next removed, so as to allow the blood to pass down into the lower part of the vessel as far as the lower permanent ligature, and, consequently, to that part of the vein which contained the poison.

"Now it must be manifest, that the solution of the poison in the blood, under such circumstances, could only act upon the system through the vessels or nerves of the *vein;* for the direct entrance of the poisoned blood into the heart, &c. was prevented by the lower ligature, and we, consequently, ought to have found, in the case of venous absorption, the effect nearly the same as that which would have occurred from the introduction of the poison to any other part of the body, in which a capillary absorption, and, consequently a greater length of time was necessary to its operation; for it will be remembered, that circulation was no longer going on through the trunk of the jugular vein itself.

"It was found, however, on the contrary, that in the space of 108 seconds after the removal of the ligature, the animal dropped in convulsions, as in the former case, and expired in three minutes and a quarter.

"The poison which was used in these two cases has never been known in dogs to produce a sensible effect upon the system, in cases of its insertion into superficial wounds of the body, in less than six minutes, and respiration usually has ceased in from a quarter of an hour to twenty-five minutes.

"Now, if we suppose that the poison of woorara produces its effect upon the system through the medium of the nerves, we can easily reconcile with that supposition the results of our two experiments: for we may attribute, in that case, the rapid operation of the poison in the last experiment, as compared with its effects upon a superficial wound of the body, to its contact with the acutely susceptible extremities of nerves distributed to the inner coat of the jugular vein itself; and we may also account for the *extreme* rapidity of its action in the first experiment, by its contact with the more extended and equally susceptible surfaces to which it must have been necessarily applied in the course of its direct circulation through the vena cava, the heart and arteries."—p. 74.

As the intimate connexion betwixt the nervous and vascular systems offer great difficulties to conclusions drawn from experiments made on either, our authors have endeavoured to obviate this impediment by attempting, as it were, to separate such connexion, and instituted experiments for the purpose.

"Two large bull-dogs, of equal size and strength, were held face to face upon a table, embracing each other, so that their breasts and necks were in contact, the animals being placed upon their sides. In this position, it will be seen that the right carotid of one dog, and the left of the other were uppermost, and that these vessels, when exposed by operation, might, while the animals were thus held together, be brought into contact. It was therefore our object to establish a connexion and circulation between these two arteries, viz. between the right carotid of one dog, and the left of the other.

"In this way, then, we established a circulation of blood from the heart of one dog, to the head of the other; and it was therefore reasonable to suppose, according to the theory of the supporters of venous absorption and cerebral contact, that the dog contributing blood to the other, would, after inoculation with a poison, be supplying his neighbor with poisoned blood, which reaching the substance of the brain, must necessarily produce the same effects in the one as in the other. This, however, was not the case; for, upon introducing the poison of nux vomica into the back of the animal, from whose carotid the blood was passing to the opposite vessel of the other dog, we found that although the usual violent effect was produced in the inoculated animal, and although that effect continued for the space of fourteen minutes, during which period a free circulation was carried on between them in the manner already mentioned, yet that not the slightest indications of the action of the poison upon the system could be observed in the other dog.

"Satisfied that the experiment had been continued long enough, the artery was then tied in the neck of the sound dog, the vessel was divided, and the sufferings of the other and expiring animal were terminated. On the following day the surviving dog continued free from all symptoms of poisoning.

"It can hardly be supposed in this case, that if the poison had been dissolved in the blood which circulated through the carotid of the poisoned dog, no portion of it would have reached the brain of the other, since it was repeatedly seen during the operation that a free current constantly passed through the vessels from one animal to the other; indeed if such had not been the case, it must be manifest that coagula would have formed in them, as well as in the brass tube by which they were connected. In the case, therefore, of sanguineous contamination and cerebral contact, we think it fair to infer that both animals ought to have been affected by the poison in nearly the same way, and at the same time, the opposite of which was proved to be the case.

"This experiment having satisfied us that the circulation of a poison through the brain was not the cause of its operation upon the body, we next proceeded to ascertain, by a nearly similar mode, whether in all cases the contamination of the blood in the veins, which supplied a poisoned part was necessary to the operation of the poison upon the system.

"Two large hounds were secured neck to neck, as in the former case, and the division and re-connexion of the jugular veins effected in a manner precisely similar to that already described in the case of the double circulation through the carotid, so that the venous blood from the head of one dog passed into the heart of the other. The animal contributing blood to the other was then inoculated on the side of the face with nux vomica, and in the usual time exhibited the usual symptoms: these continued without intermission during the space of seven minutes after the animal was first affected, the circulation being freely kept up through the artificially-connected jugulars: at the end of this period the circulation was beginning to become impeded by the formation of a small coagulum in the tube, and we therefore terminated the experiment by destroying the poisoned animal, the other dog never having shown the slightest symptoms of being poisoned. Now, if in this case the poison had been taken by the veins in a sufficient quantity to affect the distant textures to which it was carried in its course through the circulation, we do not think it possible that either dog could have escaped; at all events, if either had escaped, it would have been the poisoned animal, whose veins were prevented carrying contaminated blood from the part through the system by their connexion with those of another."—p. 86.

A double circulation was effected by connecting the lower end of the divided carotid artery of one dog with the upper end of the carotid of another, so that each animal supplied the brain of the other; and consequently the poisoned dog received from the unpoisoned animal a supply of blood equal in quantity to that with which he was parting. The form of the connexion is shewn by a plate, a rude idea of which may be conveyed by the letter X; the division of the arteries being above the superior angle, and the left side of the letter representing the carotid of one dog, the right that of the other. The experiment was as follows.

"Two large half-bred bull dogs, each weighing about forty pounds, were the animals selected for the operation. The carotid artery of each dog having been laid bare on one side, and separated from its connections with surrounding parts to the extent of three inches, temporary ligatures were applied above and below, and the arteries were divided between them, as in a former case; the brass tubes were then attached to the extremities of the vessels, and the necks of the two animals being held and closely bound together, the divided arteries were, without the least difficulty, re-connected, and the circulation reversed.

"One of the dogs was then inoculated on the back with a concentrated preparation of strychnine, which had been found upon other occasions to produce death in these animals in about three minutes and a half.

"In three minutes and a half the inoculated animal exhibited the usual tetanic symptoms which result from the action of this poison, and died in a little less than four minutes afterwards, viz. about seven minutes from the time at which the poison was inserted, during the whole of which time a free and mutual interchange of blood between the two was clearly indicated by the strong pulsation of the denuded vessels throughout their whole course.

"The arteries were next secured by ligature, and the living was separated from the dead animal; but neither during the operation, nor at any subsequent period,* did the survivor show the slightest symptom of the action of the poison upon the system.

"From these, then, and from many other similar experiments, which it would be needless to instance, we have been led to the conclusion that all poisons, and, perhaps, indeed, all agents influence the brain and general system, through an impression made upon the sentient extremities of the nerves, and not by absorption and direct application to the brain.

"So far as we are competent to judge, the conclusion is borne out by the experiments detailed in this Essay; but if those experiments be not satisfactory to our readers, we at least indulge the hope, that what has been advanced may lead to the discovery of more satisfac-

*"This animal was killed on the following day."

tory and more conclusive evidence than has yet been adduced on this truly important question; a question, indeed, of the deepest interest, not as a mere matter of curiosity, but as involving the elucidation of many of the most prominent, and at present the most mysterious phenomena of a living body.

"If found to be correct, the principle for which I contend will not be limited to the operation of those noxious agents usually denominated poisons, but it may probably tend to the better understanding both of the causes and cure of diseases in general." 91.

Such are the experiments and conclusions of our authors in proof of their theory. We have once more to apologize for devoting so much space to the review of the Essay before us, but the vast importance of the subject, and impartial justice towards the individuals at issue, absolutely required it. We think the objections and arguments employed by Dr. Addison and Mr. Morgan against those who maintain opposite opinions, very powerful, and such as require answers, if any can be given; while their experiments are ingenious and deeply interesting, their deductions in general are natural and legitimate, and their theory on the whole as well, if not better supported, than any other which has been received. We think, however, it is by no means fully established, and that much more research is necessary to entitle it to adoption. It has been contended that a nerve deprived of circulation, as the sciatic in a former experiment, could not perform its functions; but were not the nerves of the insolated jugular veins in our author's experiments in the same condition, and besides subjected to pressure for a considerable time? According to their own shewing, these nerves could not perform their functions, and it is not easy to admit, that nerves even remote in the same vessels, must not be affected by sympathy, and must not have their functions deranged by the injuries inflicted on the divided vein and surrounding parts during the experiments. The experiments upon the whole seem to us to disprove the doctrine of poisons acting by absorption. But though we think our authors have thrown much light on the action of poisons on the system, we regret that they have left the more important matter, the prevention of such action, as they found it.

